# Water content, adenylate kinase, and mitochondria drive adenylate balance in dehydrating and imbibing seeds

**DOI:** 10.1093/jxb/erx182

**Published:** 2017-07-20

**Authors:** Marie-Paule Raveneau, Abdelilah Benamar, David Macherel

**Affiliations:** 1 USC LEVA, INRA, Ecole Supérieure d’Agricultures, Université Bretagne Loire, SFR QUASAV, rue Rabelais, Angers Cedex, France; 2 IRHS, INRA, Agrocampus-Ouest, Université d’Angers, SFR QUASAV, rue Georges Morel, Beaucouzé, France

**Keywords:** Adenylate, adenylate kinase, anhydrobiosis, bioenergetics, mitochondria, oxidative phosphorylation, respiration

## Abstract

Water and life are inexorably linked, but some organisms are capable of losing almost all cellular water to enter a non-metabolic state of anhydrobiosis. This raises intriguing questions about how energy metabolism is managed during such transitions. Here, we have investigated adenylate metabolism during seed imbibition and drying using intact or fragmented pea (*Pisum sativum* L.) seeds. AMP was confirmed as the major adenylate stored in dry seeds, and normal adenylate balance was rapidly restored upon rehydration of the tissues. Conversely, re-drying of fully imbibed seeds reversed the balance toward AMP accumulation. The overall analysis, supported by *in vitro* enzyme mimicking experiments, shows that during tissue dehydration, when oxidative phosphorylation is no longer efficient because of decreasing water content, the ATP metabolic demand is met by adenylate kinase, resulting in accumulation of AMP. During seed imbibition, adenylate balance is rapidly restored from the AMP stock by the concerted action of adenylate kinase and mitochondria. The adenylate balance in orthodox seeds, and probably in other anhydrobiotes, appears to be simply driven by water content throughout the interplay between ATP metabolic demand, adenylate kinase, and oxidative phosphorylation, which requires mitochondria to be energetically efficient from the onset of imbibition.

## Teaser

AMP accumulation in dry seeds, which powers the rapid resumption of energy flow during imbibition, is driven by dehydration through the interplay between ATP demand, adenylate kinase, and oxidative phosphorylation.

## Introduction

All living organisms rely on water as the matrix of life and require a permanent energy flow to maintain homeostasis, grow, and ultimately reproduce. Interestingly, and in spite of the indispensable role of water for cellular structures and metabolism, some eukaryotes can lose almost all their cellular water and remain in the dry state for long periods, resuming life, and thus energy flow, upon the return of water. In doing so, these organisms, the so-called anhydrobiotes, enter into a state of suspended life in which structures are maintained (Keilin, 1959). Although earlier experiments suggested that some respiratory activity occurs in the dry state (e.g. Wilson and Bonner, 1971), the values reported were probably not significant. Nevertheless, it must be admitted that dry seeds are metabolically inert and, considering that respiration is arrested in the dry state, a major and intriguing question is how energy metabolism is managed by anhydrobiotes during the dehydration and rehydration periods surrounding the dry state.

Cellular energy status is often associated with the adenylate energy charge (AEC) ratio, which corresponds to the (ATP+0.5 ADP)/(AMP+ADP+ATP) ratio originally proposed to be a key metabolic modulator (Atkinson and Walton, 1967). Although the ATP/ADP ratio is often considered as the major parameter, the AEC ratio takes into account the presence of the reversible adenylate kinase (ADK), whose reaction (2ADP↔ATP+AMP) operates close to equilibrium, and thus influences the ATP/ADP ratio. In plant mitochondria, ADK is localized in the intermembrane space, and its activity strongly influences the equilibrium of free and Mg-bound adenylate pools in the cytosol and plastids (Igamberdiev and Kleczkowski, 2003, 2006). It is noteworthy that even the mechanisms of metabolic control of cellular respiration by adenylates in plants are still debated, and recent work suggested cytosolic ADP as the most important regulator of respiration (Gout *et al.*, 2014). In plants, as in other organisms, tissues with high metabolic activity well sustained by aerobic energy metabolism exhibit high AEC ratio values (Pradet and Raymond, 1983). In normoxic conditions, cells have a high AEC ratio (>0.8) because the ATP/ADP ratio is maintained high (typically 10:1) by respiration (and photosynthesis in green tissues), and thus ADK favors the production of ADP, maintaining a low level of AMP. Cells challenged with stress conditions typically have a lower AEC ratio (0.4–0.6) due to a lower ATP/ADP ratio because of high energy demand. For instance, the AEC ratio in excised maize root tips shifted from 0.9 in air to 0.6 under fermenting conditions (Saglio *et al.*, 1980). When dry seeds were investigated, low AEC ratio values were obtained, generally in the 0.1–0.4 range, which is not surprising given that dry seeds are metabolically inactive; the AEC ratio increased rapidly during imbibition to reach the typical values of metabolically active tissues (Brown, 1965; Moreland *et al.*, 1974; Abernethy *et al.*, 1977; Hourmant and Pradet, 1981; Benamar *et al.*, 2008). In agreement with the values of AEC in dry seeds, a decrease in the AEC ratio has been measured during seed development (Ching and Ching, 1972; Ching *et al.*, 1974; Quebedeaux, 1981; Kumar and Singh, 1984). A major question that has intrigued researchers since these early observations is how such dramatic changes in energy metabolism between the active hydrated and the quiescent dehydrated states could be managed. From the many studies that addressed the resumption of energy metabolism in germinating seeds, it became clear that oxygen was required for germination of almost all species and that the onset of oxygen consumption was rapid, with a systematic increase during imbibition (Parrish and Leopold, 1977; Hourmant and Pradet, 1981; Al-Ani *et al.*, 1985; Ehrenshaft and Brambl, 1990; Benamar *et al.*, 2003). Investigations into the properties of mitochondria isolated from germinating seeds revealed a general increase in activity during imbibition of oxidative phosphorylation and activation of tricarboxylic acid (TCA) cycle enzymes (Botha *et al.*, 1992; Logan *et al.*, 2001; Benamar *et al.*, 2003). From the time of the first microscopic observations of cellular ultrastructure in seeds (e.g. Bain and Mercer, 1966), it has generally been considered that mitochondria in dry seeds were dedifferentiated into simpler organelles and that extensive repair and biogenesis of the organelle was required to sustain the development of mitochondrial activities during germination (Nawa and Asahi, 1971; Solomos *et al.*, 1972; Morohashi, 1986). Recently, omics studies in Arabidopsis led to the suggestion that extensive mitochondrial biogenesis was required before the organelle resumes its bioenergetic function (Narsai *et al.*, 2011; Law *et al.*, 2012, 2014). However, regardless of these studies promoting the importance of extensive mitochondrial biogenesis for germination, it is clear that a rapid resumption of mitochondrial energy transduction is required during early seed imbibition to fuel the high cellular energy demand. What is less clear is how seed mitochondria meet this demand so quickly after they exit metabolic stasis. Here, we have explored the biochemical mechanisms that allow seed tissues to cope with the major energetic transitions that occur between the hydrated and dry states, and, reciprocally, using pea (*Pisum sativum* L. var Baccara) seeds as a model in order to provide an integrated view of the mechanisms of bioenergetics in the context of anhydrobiosis.

## Materials and methods

### Plant materials

Pea (*Pisum sativum* L. cv. Baccara) seeds were grown locally by the agronomical research institute FNAMS (Fédération Nationale des Agriculteurs Multiplicateurs de Semences, Brain-sur-l’Authion, France) and stored in sealed plastic bags at 5 °C (70% relative humidity) until required.

Pea seeds were imbibed in covered plastic (polycarbonate) boxes (18.5 × 12.5 cm×5.5 cm) in the folds of pleated blotting paper (Ref. 3236, GE Healthcare, Vélizy, France) saturated with 80 ml of deionized water. Boxes were incubated at 20 °C in the dark for the indicated time. Under these conditions, the seed lots used for the analyses started to germinate (protrusion of radicle) after 26 h of imbibition, and reached 100% germination after 72 h, with a *T*_50_ (time for 50% germination) of 43 h. For the priming treatment, seeds were imbibed for 18 h and then transferred in a box containing silica gel for drying. The water content of seeds was determined by weighing seeds before and after incubation at 95 °C for 48 h. Pea seed fragments were obtained by grinding dry seeds for 30 s with a laboratory Mixer Mill (Retsch, Haan, Germany) and sieving the ground material through a combination of 1.0, 0.7, and 0.3 mm metal grids to select fragments with size ~0.5 mm which were retained on the 0.3 mm grid. Pea seed fragments were imbibed on filter paper (Whatman Ref 1001 085, GE Healthcare) saturated with water, and placed in a Petri dish at 20 °C. Seed from barrel medic (*Medicago truncatula*) were produced in our institute. Mung bean (*Vigna radiata*), sunflower (*Heliantus annuus*), rapeseed (*Brassica napus*), and triticale (*×Triticosecale* Wittm. ex A. Camus) seeds were obtained from the FNAMS or local producers. All seed lots displayed >90% germination under standard conditions.

### Mitochondrial isolation and oxygraphy

Mitochondria were isolated from 22 h imbibed pea seeds, and oxygraphic measurements were performed at 25 °C, as described previously (Stupnikova *et al.*, 2006).

For adenylate measurements, imbibed seeds were frozen in liquid nitrogen and kept at −80 °C until extraction. Frozen seeds were maintained at very low temperature by immersion in liquid nitrogen, before grinding with steel beads using a tissue lyser (QIAGEN GmbH, Hilden, Germany). An aliquot of frozen powder (~50 mg) was taken with a liquid nitrogen pre-cooled spatula, precisely weighed (great care was taken to keep seed powder frozen at all stages) and transferred in a 1.5 ml microtube containing 400 μl of methanol/chloroform (1:1) which was immediately vortexed and incubated on ice for 10 min. After 30 s vortexing, 200 μl of H_2_O was added; the microtube tube was vortexed again and centrifuged (10 min, 13 400 *g*, 4 °C). The upper phase was withdrawn and transferred into a microtube which was stored at −80 °C until HPLC analysis. For dry seeds, grinding was performed at room temperature, and 250 μl of H_2_O were used to trigger phase separation. For isolated mitochondria, 50 μl of suspension (equivalent to 1.5 mg of protein) were projected into 400 μl of methanol/chloroform (1:1) and adenylates extracted as above.

The extracts were separated by HPLC on an IonPac AS11 column (Dionex Corp., Sunnyvale, CA, USA). Separation was performed at 30 °C, with a flow rate of 0.38 ml min^–1^. Eluent (carbonate-free KOH) concentrations were delivered by an eluent generator system (Dionex ICS-5000+EG, Thermo Fisher Scientific, Courtaboeuf, France) with a multistep concentration gradient: step 1 (0–8 min) at 17 mM KOH; step 2 (8–25 min) with a concave isocratic gradient (Dionex GP50 pump, Curve 6) from 17 mM to 100 mM; step 3 (25–30 min) at 100 mM; step 4 (30–31 min), linear gradient (100–17 mM); step 5 (31–40 min), 17 mM KOH. Compounds absorbing at 259 nm were detected with a Dionex Ultimate 3000 system (Thermo Fisher Scientific). Peaks corresponding to AMP, ADP, and ATP were identified by spiking with standard compounds (Sigma Aldrich, St Quentin Fallavier, France), and quantification was done using a standard curve of known concentrations, using the Chromeleon software (version 6.80, Dionex Corp.). Recovery of adenylate standards added after seed grinding in preliminary experiments was >95%. Three independent extractions were performed for each sample, and the results are indicated as the average and SD.

### Enzyme assays

All chemicals and enzymes were purchased from Sigma Aldrich (St Quentin Fallavier, France). The hexokinase–ADK assay was performed at 30 °C in TMK buffer (25 mM triethanolamine, 5 mm MgCl_2_, 1 mM KCl, pH 7.5) containing 2.5 mM AMP, 5 mM ADP, 2.5 ATP, 400 mM glucose, and 0.5 U of ADK (from rabbit muscle). After 10 min incubation, 10 U of hexokinase (from *Saccharomyces cerevisiae*) were added and the reaction was allowed to proceed. The pyruvate kinase–ADK assay was performed at 30 °C in TMK buffer containing 20 mM AMP, 10 μM ADP, 0.5 U of ADK (from rabbit muscle), and 10 U of pyruvate kinase (from rabbit muscle). After 10 min incubation, 20 mM phosphoenolpyruvate was added and the reaction was allowed to proceed. For both enzyme assays, aliquots (10 μl) were withdrawn at specific time points, and enzymes were inactivated by heating microtubes containing the samples at 90 °C for 5 min and cooling down on ice. Samples were diluted with 0.5 ml of ice-cold H_2_O and centrifuged (10 000 *g*, 10 min, 4 °C). A 10 μl aliquot of the supernatant was directly injected in the HPLC system for adenylate analysis.

### Statistical analysis

Statistical analysis was performed for all data using the R software (Version 3.2.4, http://www.R-project.org/). Comparisons of means with appropriate parametric (*t*-test, ANOVA) or non-parametric (Wilcoxon, Kruskal–Wallis) tests were used to estimate the statistical significance of the variations indicated in the text.

## Results

### Adenylates in dry seeds and during imbibitions

**Fig. 1.**
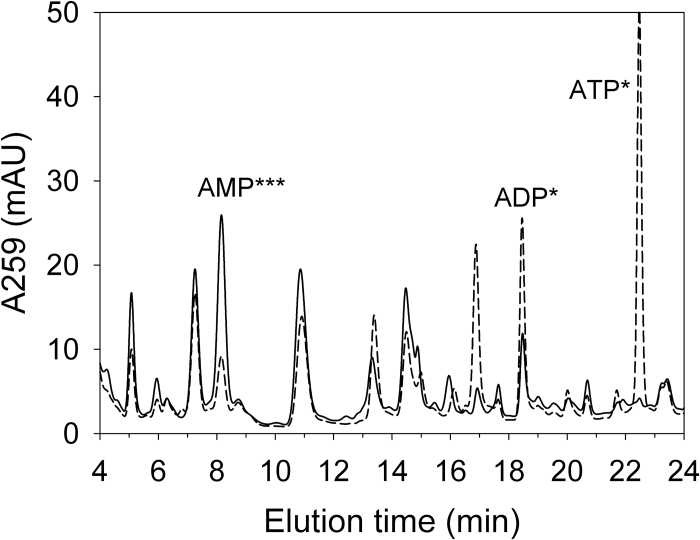
HPLC separation of dry and imbibed pea seed extracts. The representative chromatograms illustrate the separation of adenylates and other UV-absorbing unidentified compounds in the extracts of dry seeds (solid line) or 16 h imbibed seeds (dashed line). A comparison of means between the dry and 16 h imbibed seed extracts indicated significant differences for AMP (*t*-test, ***P-value=0.0002344), ADP, and ATP (Wilcoxon test, *P value=0.03125).

The extraction and analysis procedure allowed clear separation by HPLC of AMP, ADP, and ATP which were identified by spiking. Examples of chromatograms shown in Fig. 1 illustrate the sharp contrast between the adenylate profiles of the dry and imbibed (16 h) seed extracts. Imbibed seeds display a classical adenylate profile with a major peak for ATP, a lower one for ADP, and a small peak for AMP. In the dry seed extract, the peak of AMP is the highest, while the peak of ADP is lower, and the peak of ATP has almost disappeared. Therefore, AMP appears to be the major adenylate in dry pea seeds, which contain only traces of ATP, and some ADP. This confirms the expected difference between the adenylate content of dry and imbibed pea seeds that was reported many years ago (Brown, 1965). Indeed, using the same extraction method, AMP was found to be the major adenylate in the dry seeds of 16 different cultivars of pea (data not shown), but also in dry seeds from different species of various families (*Fabaceae*, *Brassicaceae*, *Asteraceae*, and *Poaceae*) (Table 1).

**Table 1.** Adenylate content in dry seeds from different species. Adenylates were extracted from dry seeds and quantified by HPLC The values (nmol g^−1^ DW) are indicated with their SD (*n*=3).

Species (family)	AMP	ADP	ATP
*Pisum sativum* (*Fabaceae*)	117.50 ± 8.43	33.83 ± 4.22	4.27 ± 0.59
*Medicago truncatula* (*Fabaceae*)	140.43 ± 28.97	37.63 ± 1.86	29.21 ± 2.73
*Vigna radiata* (*Fabaceae*)	114.49 ± 12.33	50.50 ± 5.19	11.76 ± 4.43
*Helianthus annuus* (*Asteraceae*)	83.67 ± 1.52	9.21 ± 1.66	22.54 ± 4.22
*Brassica napus* (*Brassicaceae*)	110.75 ± 9.80	6.36 ± 0.88	20.20 ± 4.98
*×Triticosecale* Wittm. ex A. Camus (*Poaceae*)	8.57 ± 0.36	1.63 ± 0.24	0.80 ± 0.62

A comparison of means for all species with a Kruskal–Wallis test (α=0.05) indicated that the AMP amount was significantly different from those of ADP (*P*-value=3.9 × 10^–5^) and ATP (*P*-value=4.5 × 10^–5^), while ADP and ATP amounts were not significantly different (*P*-value=0.137).

**Fig. 2.**
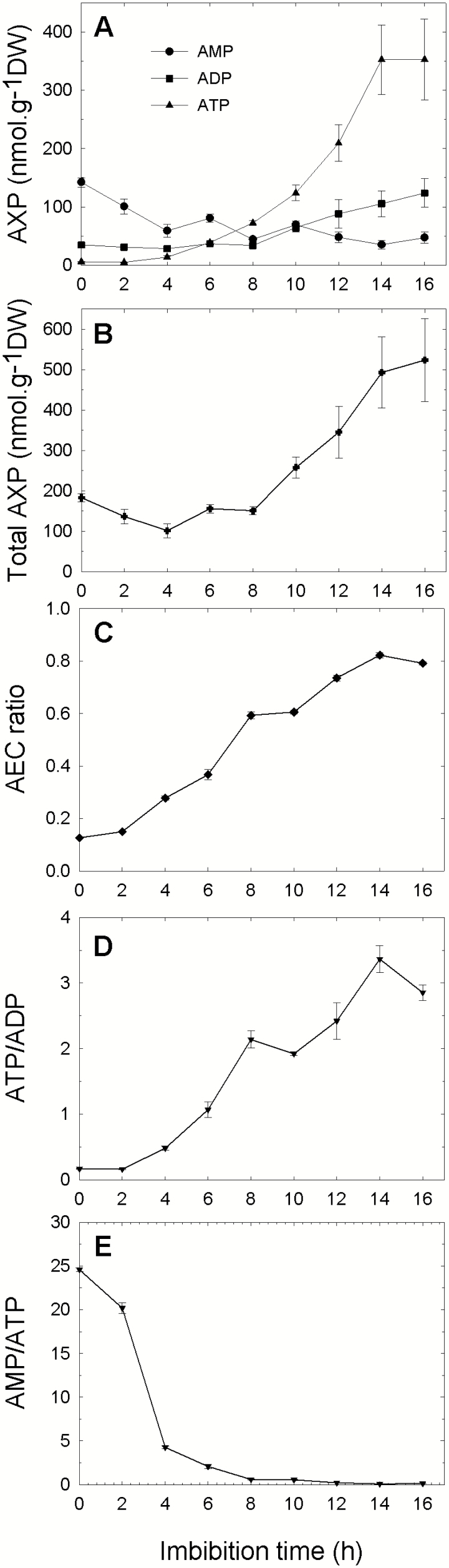
Time course of adenylate species content during pea seed germination. Seeds were allowed to imbibe in water, at 20 °C, for different times. Adenylates were extracted and quantified by HPLC. The upper graph (A) shows data for AMP (circle), ADP (square), and ATP (triangle). The total adenylate content is shown in (B); (C–E) correspond to the indicated adenylates ratio. AEC is the adenylate energy charge ratio [(0.5 ADP+ATP)/(AMP+ADP+ATP)]. Values are indicated with their SD (*n*=3 biological replicates).

A time course experiment (Fig. 2A) clearly shows a progressive and large increase in ATP which reaches its maximum level after 14 h of imbibition. AMP decreases gradually, while ADP shows a moderate increase starting after 8 h of imbibition. The total amount of adenylates is almost constant up to 8 h of imbibition, before increasing >3-fold (Fig. 2B). These results suggest that during the first hours of seed imbibition, interconversion occurs between the three adenylates, and then the adenylate pool size is increased by *de novo* biosynthesis. Figure 2C shows that the AEC ratio was very low in the dry seed extracts (<0.2), but progressively increased up to 0.8 (after 14 h of imbibition), a value well in agreement with that of tissues with active metabolism. The ATP/ADP ratio, another indicator of energy status, was very low in dry seed extracts, but increased progressively to high values (Fig. 2D), as a consequence of the large increase in ATP concentration. Since AMP is by far the most abundant adenylate in dry and quiescent seeds, and ATP is the major adenylate in imbibed seeds with high energy metabolic activity, the AMP/ATP ratio appears to be an indicator of the transition between anhydrobiosis and the active state. Indeed, this ratio shows a rapid decrease during the first hours of imbibition (Fig. 2E). As a whole, the changes in adenylate concentrations during early imbibition agree well with the resumption of seed respiration and metabolism, which are accompanied by a necessary readjustment of the AEC ratio, the major changes being the decrease in AMP and increase in ATP.

**Fig. 3.**
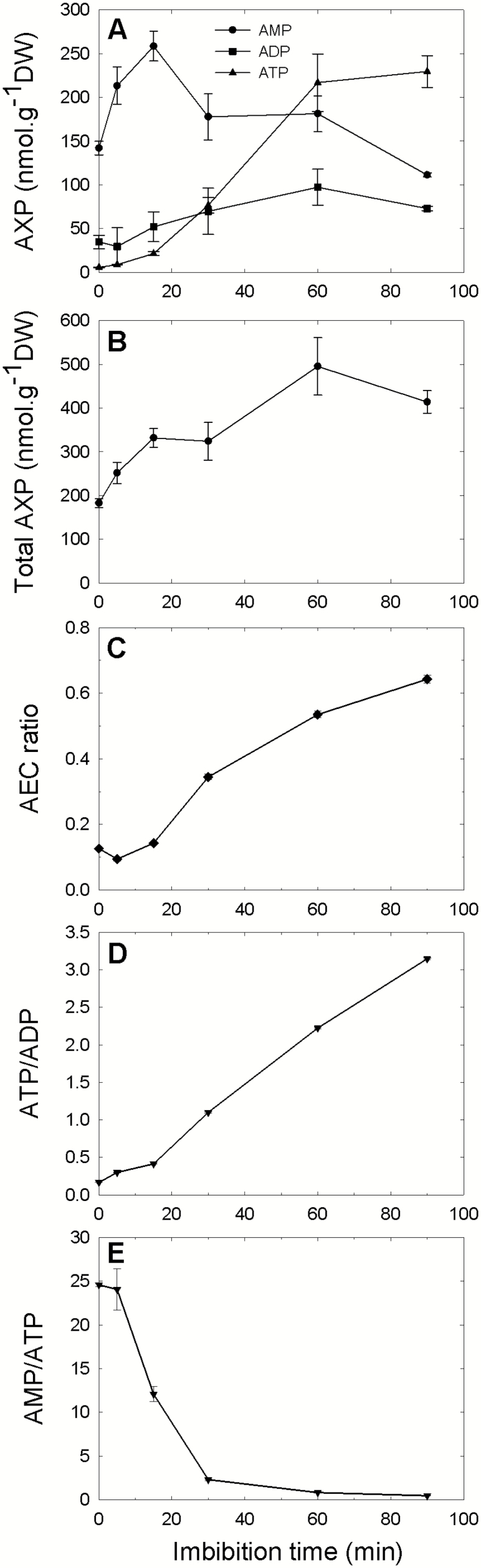
Time course of adenylate species content during imbibition of pea seed fragments. Dry pea seed fragments (~500 μm) were allowed to imbibe in water at 20 °C for different times. Adenylates were extracted and quantified by HPLC. The upper graph (A) shows data for AMP (circle), ADP (square), and ATP (triangle). The total adenylate content is shown in (B); (C–E) correspond to the indicated ratio of adenylates. AEC is the adenylate energy charge ratio [(0.5 ADP+ATP)/(AMP+ADP+ATP)]. Values are indicated with their SD (*n*=3 biological replicates).

However, these results do not provide a very precise view of the events occurring during tissue rehydration because, due to their size, the pea seeds used in this study required ~6 h to reach full imbibition of their tissues. Thus, the adenylates extracted from seeds come from a mix of cells with variable water content and/or having experienced different durations of hydration. To gain a better view of the variation in adenylate content during early imbibition, we used dry seed fragments of ~0.5 mm which fully imbibe in <10 min on wet filter paper. Since the embryo axis represents only 2–3% (w/w) of the dry seed mass, and integuments were eliminated in the sieving process, the fragments originate essentially from cotyledon tissues. Their ATP content showed a rapid increase during the experiment, reaching values >200 nmol g^−1^ after 60 min of imbibition (Fig. 3A). In comparison, >6 h of imbibition were required to reach a similar ATP content when using intact seeds (see Fig. 2A), which reflects well the heterogeneity of the seed with respect to water status. ADP content in the fragments showed a moderate increase up to 60 min of imbibition (Fig. 3A). Surprisingly, AMP content displayed a rapid increase from 150 nmol g DW^−1^ to 260 nmol g DW^−1^ during the first 15 min, before steadily decreasing, as expected from the time course measured in whole seeds. Since this increase in AMP concentration occurred very quickly (+70% within 5 min of imbibition), it was suspected to be an extraction artifact, and not the result of a metabolic event. We therefore analyzed the variation in adenylate contents during imbibition of seed fragments at 0 °C, a temperature at which metabolic reactions are expected to be much slower than at 20 °C. At 0 °C, there were no detectable increases in ATP or ADP content, while the AMP content increased from 150 nmol g DW^−1^ to 230 nmol g DW^−1^ over the first 15 min of imbibition but remained stable thereafter (see Supplementary Fig. S1 at *JXB* online). These results confirm that the general increases in ATP and ADP, or the decrease in AMP content are of metabolic origin, which is not the case for the initial transient increase in AMP, which occurs similarly at 0 and 20 °C (Supplementary Fig. S1). This transient increase in AMP is therefore probaby attributable to a better extractability from hydrated than from dry material, and not from a metabolic process. Overall, the change in adenylate concentrations in imbibing seed fragments occurs much faster than in whole seeds, but it follows a similar pattern, with a major increase in ATP, a moderate increase in ADP, and a decrease in AMP content. These trends are well reflected by the fast increase in AEC and ATP/ADP ratios, or the fast decrease in the ATP/AMP ratio (Fig. 3C–E), which demonstrate that dry seed tissues restore their energy balance very rapidly upon rehydration. Interestingly, the total amount of adenylates progressively increased during the first hour (Fig. 3B), suggesting that the rapid resumption of metabolism which was observed with the seed fragments was accompanied by a *de novo* synthesis of adenylates. In the case of whole seed imbibition, *de novo* synthesis of adenylates was only apparent after several hours of imbibition, namely when most seed tissue had regained an active metabolism.

### Dehydration triggers AMP accumulation

**Fig. 4.**
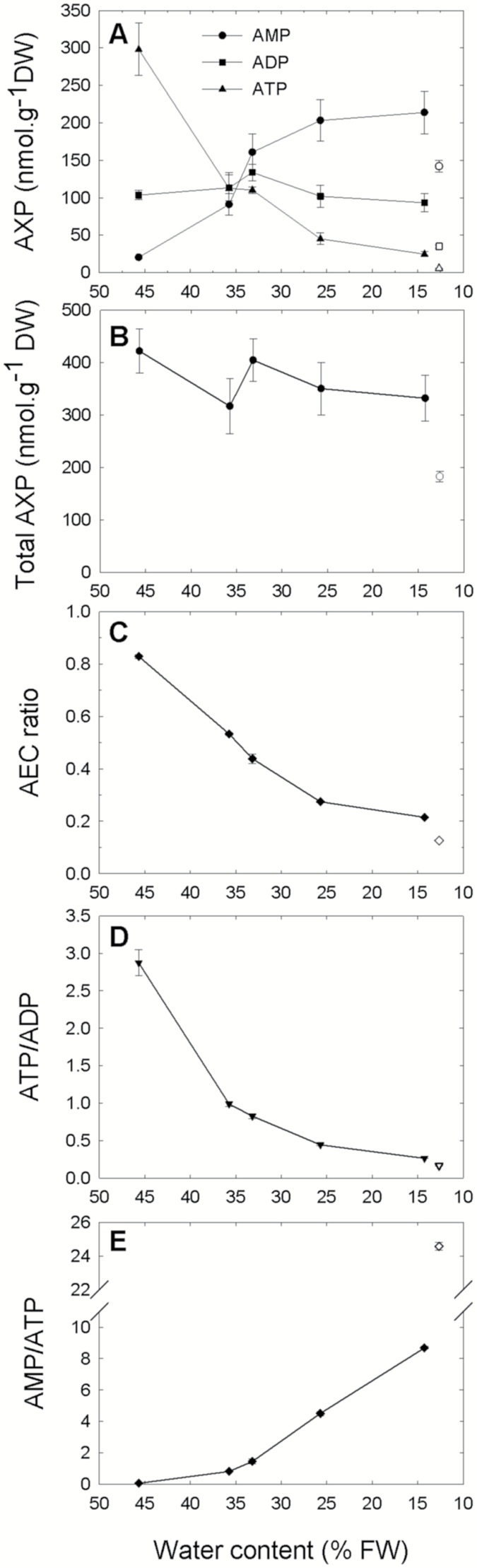
Time course of adenylate species content during dehydration of imbibed pea seeds. Dry pea seeds imbibed in water at 20 °C for 18 h were dried on silica gel. Samples were analyzed for water content and adenylate contents after 0, 2, 4, 7, and 24 h of drying. Their respective water contents were 45.6, 35.7, 33.2, 25.7, and 14.2% (fresh weight basis).The upper graph (A) shows data for AMP (filled circle), ADP (filled square), and ATP (filled triangle) as a function of decreasing water content during dehydration. The open symbols indicate the original adenylate contents of dry seeds before imbibition and dehydration. (B) The total adenylate ratio; (C–E) correspond to the indicated ratio of adenylates, also expressed as a function of water content during dehydration. Open symbols indicate the original values of adenylate contents for dry seeds before the experiment. AEC is the adenylate energy charge ratio [(0.5 ADP+ATP)/(AMP+ADP+ATP)]. Values are indicated with their SD (*n*=3 biological replicates).

In dry pea seeds, 75% of the extractable adenylate pool was attributed to AMP, which is expected to accumulate during late development of seeds, probably during final desiccation, which brings metabolism to a complete arrest (quiescence) due to the lack of cellular water. An intriguing question is whether dehydration is sufficient to trigger this major change in the adenylate pool, as it applies major constraints on cell components and metabolism. Since natural desiccation during pea seed development is a rather slow process, we took advantage of the fact that orthodox seeds remain desiccation tolerant during germination ( i.e. until radicle protrusion) to dehydrate imbibed seeds rapidly and analyze their adenylate content during the process. Pea seeds were thus allowed to imbibe for 18 h, and then they were dehydrated on silica gel for 24 h, which brought them back close to their initial dry seed water content. This procedure is similar to the priming treatments used in seed technology for selected species in order to improve the seed germination rate and homogeneity, and such a priming treatment has been shown to be effective with pea seeds (Benamar *et al.*, 2003). This treatment resulted in an almost complete reversal of the situation occurring during dry seed imbibition (Fig. 4A). As dehydration proceeded, there was little change in ADP content, but the ATP content dropped, and that of AMP increased markedly (Fig. 4A). Since there was no significant variation in the total adenylate content during dehydration (Fig. 4B), the AMP accumulation probably proceeds at the expense of ATP.

Interestingly, when the seeds were dried back close to their initial water content, they contained higher amounts of each adenylate (Fig. 4A, B). This enrichment in adenylates is due to the increase in the adenylate pool size occurring during imbibition (Fig. 2B), and could possibly be involved in the higher germination performance of primed seeds. During dehydration, AEC and ATP/ADP ratios decreased rapidly, illustrating the drop in cellular energy status induced by dehydration (Fig. 4C, D). As water content initially decreased from 45% to 35%, the ATP/AMP ratio started to increase slowly, then it increased rapidly and steadily until the end (Fig. 4E). Overall, these results show that dehydration triggers compositional modification of the adenylate pool toward the accumulation of AMP at the expense of ATP, resulting in a strong accumulation of AMP in dry seeds.

### Adenylate kinase is a major player in anhydrobiosis

The strong accumulation of AMP occurring in dry seeds suggests the following hypothesis. During dehydration, the drop in water content will hamper, at some point, the proper functioning of mitochondria, which are normally the main ATP providers through the complex process of oxidative phosphorylation (OXPHOS). However, there is still a high demand for ATP by the energy-consuming systems that remain operational until the water content drops further. The resulting accumulation of ADP would drive ADK to provide ATP and AMP, with the former being immediately converted into ADP by energy-demanding systems, and the whole process resulting in inevitable AMP accumulation. To test the hypothesis, a simple *in vitro* system was established to mimic ATP consumption (using hexokinase and glucose) and ADK function during dehydration, at a stage when OXPHOS would no longer be operational, with the objective of measuring the composition of the adenylate pool directly using HPLC quantification. Equivalent amounts of AMP, ADP, and ATP were first mixed with ADK and a large amount of glucose, and, after 10 min of incubation, there was no apparent change in the proportion of adenylates (Supplementary Fig. S2), which indicates that the system was stable. Then, hexokinase was added, allowing the rapid conversion of glucose into glucose 6-phosphate and the consumption of ATP. The time course analysis after hexokinase addition clearly demonstrates the rapid conversion of almost the entire adenylate pool into AMP (Supplementary Fig. S2). Although the results of such *in vitro* experiments are predictable based on biochemical parameters, they clearly, nonetheless, illustrate how rapidly and efficiently the adenylate pool can be converted into AMP by ADK in dehydrating seeds. It is noteworthy that ADK, which can be lyophilized, and therefore unaffected by drying, is thus expected to operate as long as enough free water is available during seed dehydration.

Conversely, as was postulated by Raymond *et al.* (1987), ADK could also have a major role in the restoration of adenylate equilibrium during the rapid hydration that occurs during seed imbibition, as was shown with pea seed fragments. In this case, the ADK reaction would be driven in the direction of ADP formation in order to recycle the abundant stock of AMP, but this requires ATP which is in high demand to fuel reviving metabolism. However, because of the relatively high amount of AMP and low amount of ADP available at the onset of imbibition, any ATP available would be converted with one AMP by ADK into two ADPs. Thus, as soon as the rehydrated mitochondria are able to produce ATP by OXPHOS, the AMP stock will be used rapidly by ADK, restoring the normal equilibrium of adenylates in hydrated and active tissues. To mimic the restoration of the adenylate pools by ADK during rehydration, we set up another *in vitro* assay in which the resumption of OXPHOS was simulated by the activity of pyruvate kinase producing ATP by substrate-level phosphorylation. In the initial state, the reaction mixture contained a high amount of AMP, a trace of ADP, ADK, and pyruvate kinase, and remained in a stable state until the addition of phosphoenolpyruvate, that triggered the initial formation of ATP, leading to rapid re-equilibration of the adenylate pools (Supplementary Fig. S2). Again, this *in vitro* experiment, albeit predictable, confirms the efficiency and rapidity of ADK in providing ADP for an ATP-generating system, as in the case of imbibing seeds.

**Fig. 5.**
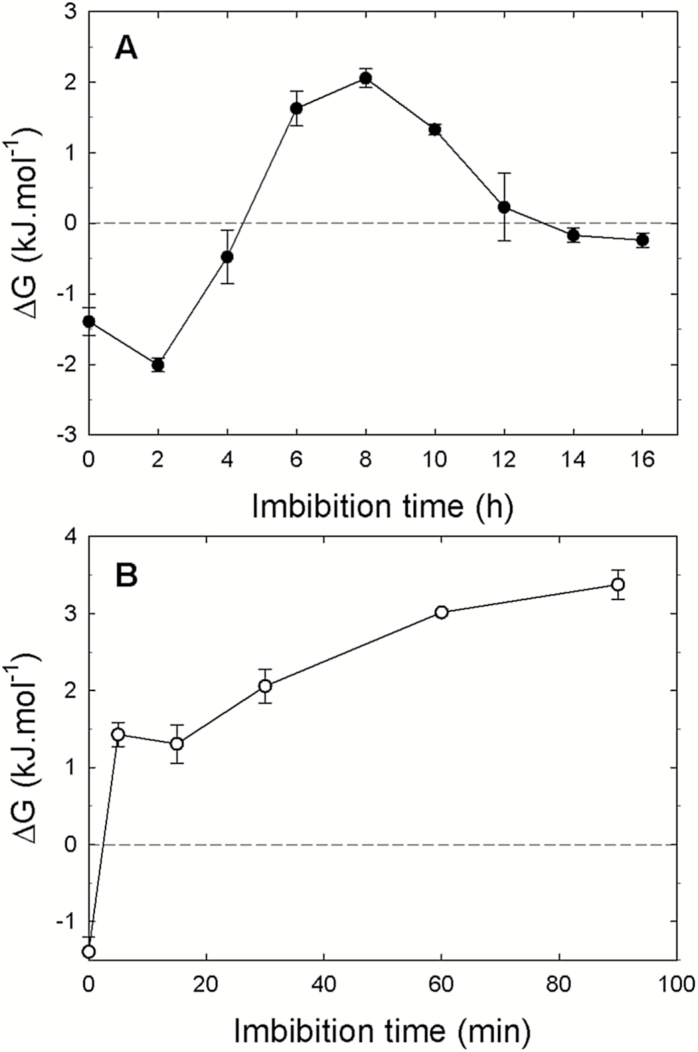
Evolution of apparent Gibbs energy for adenylate kinase reaction during imbibition of intact seeds and pea seed fragments. Intact dry pea seeds (A) or seed fragments (B) were allowed to imbibe at 20 °C for different times. The apparent Gibbs energy (Δ*G*) for the adenylate kinase reaction (2ADP↔AMP+ATP) was calculated from the adenylate contents using a *K*_eq_ value of 1.2.

In an attempt to correlate the measured amounts of adenylates during seed imbibition with thermodynamic parameters of ADK, the apparent Gibbs energy of the ADK reaction (2 ADP→AMP+ATP) was calculated using a value of 1.2 for *K*_eq_ (Canelas *et al.*, 2011). In the case of whole seed extracts, low Δ*G* values (between –2 kJ mol^−1^ and 2 kJ mol^−1^) were measured during germination (Fig. 5A), which agrees well with the expected reversibility of the reaction catalyzed by ADK. The observed evolution of Δ*G* suggests that the reaction would proceed in the AMP+ATP→2ADP direction for up to 4 h of imbibition, then in the opposite direction up to 12 h of imbibition, and finally reaching an almost perfect equilibrium (Fig. 5A). These results do not fit with a model that would favor production of ADP during early imbibition, but their interpretation should be treated with caution, in particular because of the heterogeneity of seed tissues in terms of imbibition that was highlighted previously, but also because the Δ*G* values are low. Indeed, when the analysis was performed using seed fragments that imbibe rapidly, there was a very fast rise in Δ*G*, which then continued to increase slowly (Fig. 5B). In this case, the tissues are homogenous in term of hydration, and the measured Δ*G* probably reflects better the thermodynamic force that could drive the ADK reaction at the tissue level, and it agrees well with the model in which ADK activity operates in the 2ADP→AMP+ATP direction during early imbibition.

### Seed mitochondria are self-sufficient in restoration of the adenylate pools from AMP

**Fig. 6.**
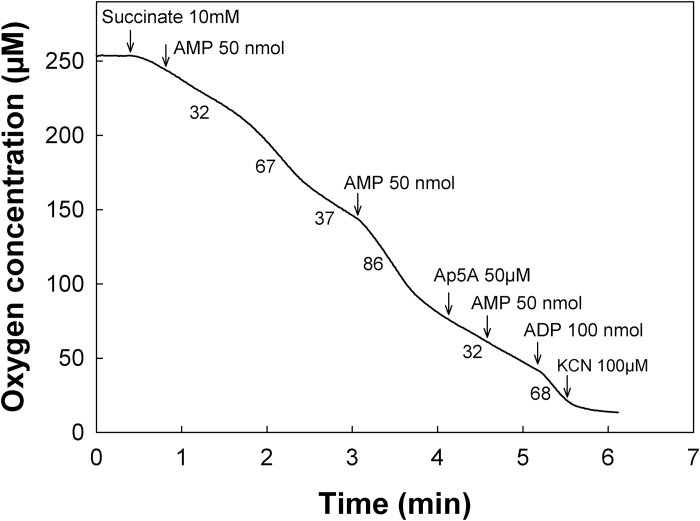
AMP-dependent oxidative phosphorylation by pea seed mitochondria. The graph shows the oxidation of succinate by intact mitochondria isolated from pea seeds. Arrows indicate the addition of the various compounds, and numbers under the traces refer to the rates of oxygen consumption in nmol min^−1^ mg^−1^ protein. Ap5A, P1,P5-di(adenosine-5′) pentaphosphate.

When cells become hydrated in the tissues of imbibing seeds, there is an urgent need to revive energy metabolism, and thus to restore mitochondrial respiration and an appropriate AEC ratio, and, indeed, mitochondrial oxygen consumption can be detected after a few minutes of imbibition of pea seed fragments (Benamar *et al.*, 2003). Since it has been shown earlier that isolated potato tuber mitochondria could perform OXPHOS with AMP as the sole source of adenylate (Roberts *et al.*, 1997), we examined whether pea seed mitochondria would display a similar capacity, which would allow the organelle to restore the adenylate balance rapidly upon cellular rehydration. It is noteworthy that pea seed mitochondria were already known to harbor relatively high ADK activity that has been used as a marker of the intermembrane space (Grelet *et al.*, 2005). Indeed, when pea seed mitochondria oxidizing succinate in state 4 were supplied with 50 nmol AMP, a transient increase in oxygen consumption rate was observed after a lag time of a few minutes (Fig. 6). This indicates that AMP must have been converted into ADP, which was then phosphorylated by OXPHOS (state 3 respiration), provoking a decrease in the proton gradient and concomitant increase in electron transfer and oxygen consumption. Interestingly, a second addition of AMP induced a similar transition to state 3, but without a lag time, indicating that AMP was then immediately converted to ADP. The addition of P1,P5-di(adenosine-5') pentaphosphate (Ap5A), an inhibitor of ADK (Feldhau *et al.*, 1975), prevented further stimulation of oxygen consumption by AMP, but not by ADP, confirming the involvement of ADK in the production of ADP for OXPHOS. A similar lag time after the first AMP addition was reported previously for potato tuber mitochondria, and interpreted in a manner that is probably also applicable to pea seed mitochondria (Roberts *et al.*, 1997). The lag time after the first AMP addition would correspond to the ‘mobilization’ of initial amounts of ATP from internal stores to prime the ADK reaction in the intermembrane space, yielding the first ADP molecules. These are then phosphorylated through OXPHOS, and this ATP would in turn feed the ADK reaction to exhaust the entire available AMP pool rapidly. On the second addition of AMP, the absence of a lag time is explained by the fact that ATP is now available, thanks to the conversion of the entire initial amount of AMP into ATP by the ADK–OXPHOS combination. To confirm the presence of internal stores of adenylates in pea seed mitochondria, their amounts were determined by HPLC using extracts from organelles purified from 22 h imbibed seeds. Purified pea seed mitochondria were found to contain 23, 107, and 208 pmol mg protein^−1^ of AMP, ADP, and ATP, respectively. This shows that mitochondria, even after the long isolation procedure, still maintain internal stores of adenylates, and in particular of ATP, that could be ‘mobilized’ for priming the ADK reaction to produce ADP. Although it is not possible to extract intact mitochondria from dry seeds, and thus to analyze their adenylate content, it is very likely that even though the ATP amount is low in dry seeds, traces of ATP remain sequestered in mitochondria.

## Discussion

**Fig. 7.**
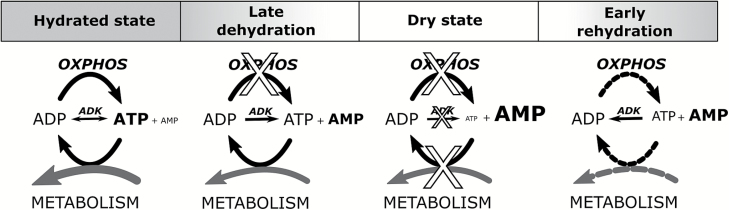
Energy metabolism in the context of anhydrobiosis. In the hydrated state, ATP production is sustained by oxidative phosphorylation (OXPHOS) to fuel the energy demand of metabolism. Adenylate kinase (ADK) contributes to the balance of adenylates when needed. During late dehydration, it is postulated that OXPHOS activity is blocked at a stage where many metabolic reactions are still active. This shifts the ADK equilibrium toward ATP production, forcefully leading to the accumulation of AMP in the dry seeds, which are metabolically inactive. During early rehydration, traces of ATP allow the initial production of ADP by ADK, and the resumption of OXPHOS allows a rapid use of the AMP pool with the help of ADK.

The results presented here provide a framework for the control of energy metabolism and adenylate pools in the context of desiccation tolerance, with the accumulation of AMP in the dry state as a pivotal event (Fig. 7). As mimicked in an *in vitro* system, the accumulation of AMP in dehydrating tissues simply results from a shift in equilibrium of ADK toward ATP production at a time during dehydration when mitochondria are no longer able to generate ATP, which is nevertheless still required by many cellular processes. Inevitably, this will lead to the accumulation of AMP, which becomes the adenylate storage form in the dry state, explaining the low energy charge ratio typical of dry seeds (Al-Ani *et al.*, 1985). During early imbibition, ADK would first operate in the AMP+ATP→2ADP direction, which fits with apparent thermodynamic data measured during seed fragment imbibition, thus providing ADP for OXPHOS and leading to rapid re-equilibration of the adenylate pools. Then, during late imbibition of whole seeds, there is an increase in the total amount of adenylates which probably reflects a general increase in metabolic activities in preparation for germination. Such an increase could result from *de novo* biosynthesis or from the purine salvage pathway which recycles adenosine released by degradation of nucleotides and reactions related to methionine and *S*-adenosylmethionine metabolism (Stasolla *et al.*, 2003). Although further studies would be needed to explain this late increase of the total adenylate pool, it is noteworthy that in Arabidopsis methionine biosynthesis appeared essential during germination (Gallardo *et al.*, 2002) while high amounts of methionine also accumulated during seed development (Frank *et al.*, 2015). Interestingly, earlier work on a model of re-induction of desiccation tolerance in germinating radicles revealed that desiccation-tolerant radicles had a lower rate of respiration than their desiccation-sensitive counterparts (Leprince *et al.*, 2000). Such controlled repression of respiration was thus proposed to prevent imbalance of metabolism and resulting oxidative stress, and thus to be an important contribution to desiccation tolerance. Whether a similar phenomenon occurs during dehydration of seeds has not been established, but it would certainly contribute to the accumulation of AMP.

Reversing the trend during rehydration of tissues is more challenging, because ADK will require a sustained input of ATP to make use of the AMP store to regenerate ADP, and thus mitochondria must be operational immediately upon rehydration to provide the required ATP to restore the adenylate pools. It is therefore not surprising that pea seed mitochondria were found to accumulate protective proteins such as HSP22, a small heat shock protein protein, and LEAM, a late embryogenesis abundant protein (Bardel *et al.*, 2002; Grelet *et al.*, 2005). Such proteins are expected to contribute to the protection of mitochondria during desiccation. Indeed, LEAM was shown to be a matrix-localized intrinsically disordered protein that upon dehydration folded into a helical form to insert laterally into the inner membrane to provide protection in the dry state (Tolleter *et al.*, 2007, 2010). It is therefore conceivable that most seed mitochondria emerge from the dry state with sufficient membrane intactness to oxidize rapidly substrates such as NADH and succinate, a process that does not require complex TCA cycle machinery, in order to restore the adenylate pools from the AMP store, and sustain energy transduction during germination, including the energy needs for biogenesis. There is little doubt that sufficient amounts of respiratory substrates [sugars, organic acids, and NAD(P)H] remain in the dry seed and can fuel mitochondria upon rehydration, as testified by the rapid resumption of oxygen consumption by seeds, embryo, or tissues (Ehrenshaft and Brambl, 1990; Logan *et al.*, 2001; Benamar *et al.*, 2003). However, whether or not dry seeds harbor energy-competent mitochondria has been challenged by the concept of de-differentiation occurring during late seed development, leading to pro-mitochondria that require extensive biogenesis of their component parts during imbibition in order to restore bioenergetic function (Narsai *et al.*, 2011; Law *et al.*, 2012, 2014). Biogenesis of mitochondrial proteins is certainly essential during germination, not only to ensure organelle maintenance, but also to cope with the developmental program of the embryo, from germination to the establishment of a photosynthetic autotrophic seedling. However, the first critical step for rehydrating seed tissue must be the rapid establishment of cell metabolism in its entirety to fuel cellular repair and maintenance in addition to anabolic and catabolic metabolism (Al-Ani *et al.*, 1985; Macherel *et al.*, 2007). This was recently confirmed by the observation of competent mitochondria in dry Arabidopsis seeds which were able to regain a membrane potential within minutes of hydration (Paszkiewicz *et al.*, 2017).

Isolated pea seed mitochondria were shown to be able to produce ATP directly from AMP thanks to their ADK localized in the intermembrane space, and to an internal store of ATP to prime the process. Interestingly, an AMP/ATP transporter localized in the inner membrane has been characterized in Arabidopsis mitochondria (Palmieri *et al.*, 2008). Such a transporter would facilitate the initial extraction of traces of ATP stored in the matrix space to prime the ADK reaction in the intermembrane space. ADK thus appears to play a crucial role to build, and later use, the huge AMP pool which appears as a signature of the dry state in seeds. The enzyme is a ubiquitous and reversible phosphotransferase contributing to the homeostasis and biosynthesis of adenylates, generally present as multiple isozymes in multicellular organisms (Dzeja and Terzic, 2009). It must be stressed that ADK, which is a small polypeptide in the 20 kDa range, is probably unaffected by drying since several enzymes from animal sources (myokinase) are sold lyophilized. A question that can be raised is whether dedicated isoforms are involved in the context of anhydrobiosis. Unfortunately, the question cannot easily be currently addressed in pea, or even in *M. truncatula*, because of insufficient data about the identification, localization, and expression of isoforms. We therefore searched for evidence of ADK gene expression in Arabidopsis, where seven genes (*AMK1*, *At2g37250*; *AMK2*, *At5g47840*; *AMK3*, *At5g50370*; *AMK4*, *At5g63400*; *AMK5*, *At5g35170*; *AMK6*, *At2g39270*; and *AMK7*, *At3g01820*) were identified by a phylogenetic analysis (Lange *et al.*, 2008). Subcellular predictions and localization experiments with green fluorescent protein (GFP)) fusions as well as evidence from published proteomics data suggested that while AMK2 and AMK5 are clearly localized to plastids, other isoforms are mitochondrial or dual targeted to mitochondria and plastids (Lange *et al.*, 2008). For six of these genes (*AMK1–AMK5* and *AMK7*) for which transcriptomic data are available on the Arabidopsis eFP browser (Winter *et al.*, 2007), levels of expression in different organs were found to be quite variable but substantial, in agreement with the housekeeping role of ADK (Supplementary Fig. S3). The levels of transcripts for the six genes are low in dry seeds, but for four of them (AMK1–AMK4) a major increase is observed after 24 h imbibition. A closer examination of the transcript levels during seed development reveals that *AMK2–AMK4* transcripts were 2- to 4-fold more abundant during early seed development than in dry mature seeds (Supplementary Fig. S3). In particular, the *AMK4* transcript level remains high until the last stage, before seed desiccation. Mining for proteomics data obtained from analysis of different organs of Arabidopsis confirms that for six of the genes (no data for AMK7), proteins could be detected with variable levels of expression (Supplementary Fig. S3). Three ADKs were detected in dry seeds (AMK3–AMK5) and, in addition, the isoform AMK4 has been identified in the mitochondrial proteome of an Arabidopsis cell culture (Heazlewood *et al.*, 2004). Although in-depth molecular and genetic approaches would be required, if feasible, to determine the role of AMK4 in seeds, these expression data show that ADKs targeted to mitochondria are probably expressed in Arabidopsis seeds, and this fits with the biochemical model presented here. Conversely, it would be very difficult to perform a similar biochemical study on Arabidopsis because of the large amount of material needed (a single pea seed weighs as much as 12 000 Arabidopsis seeds).

The accumulation of AMP in dry seeds imposes considerable variations in the adenylate contents and ratios during desiccation and imbibition, which could possibly serve as signals for controlling energy metabolism. In eukaryotes, AMP-activated protein kinases (AMPKs) are critical energy sensors that up-regulate the energy production process and limit anabolic pathways to restore the energy status when it is compromised (e.g. by nutrient deprivation; Ghillebert *et al.*, 2011). AMP, as an allosteric activator, was classically considered as the main signal controlling mammalian AMPK, but recent work suggests that ADP and AMP also trigger activation of the enzyme by promoting phosphorylation of a threonine residue in the catalytic site, providing a finer control of AMPK as a function of the energy charge (Oakhill *et al.*, 2012). In plants, SnRK1, the homolog of AMPK, has major roles in the regulation of global metabolism, development, and stress response (Polge and Thomas, 2007; Crozet *et al.*, 2014; Broeckx *et al.*, 2016). However, a detailed characterization of Arabidopsis SnRK1 revealed distinctive structural features and a lack of regulation by AMP and ADP (Emanuelle *et al.*, 2015). It is therefore likely that SnRK1 is not involved in the control of energy metabolism in the context of anhydrobiosis and, indeed, the evolution of adenylate ratios (AMP/ATP or ATP/ADP) does not match with the trends of respiratory activities which decline during desiccation and sharply increase during imbibition. A deeper knowledge about the function of AMPK/SnRK1 in plants will therefore be required to clarify a possible role, if any, for these energy sensors in the context of anhydrobiosis. Indeed, the large fluctuations in adenylates occurring during desiccation or early imbibition appear to be essentially imposed by the interplay between ADK, OXPHOS, and ATP-consuming reactions, and the relative capacities of these systems depend mainly on cellular water content rather than on sophisticated regulatory systems. Recently, high-throughput measurement of single seeds revealed that respiration of imbibing seeds of several species was highly sensitive to water potential (Bello and Bradford, 2016). An open question is therefore whether respiration could be directly controlled by water-dependent changes in adenylate balance. However, considering that adenylate kinase activity should not be affected by physiological changes in water potential, it is more likely that the overall respiratory metabolism, including OXPHOS, is depressed by changes in water potential. Indeed, many diverse effects of water stress on plant respiration and mitochondria have been reported, but there is still limited understanding of how respiration is controlled under these conditions (Atkin and Macherel, 2009).

The overall mechanisms we described are likely to occur in most if not all orthodox seeds. An intriguing question is whether or not other anhydrobiotic eukaryotes use a similar strategy to manage their energy metabolism throughout the dry state. Interestingly, AMP was also found to be the major adenylate in dry encysted embryos of the brine shrimp *Artemia franciscana*, an anhydrobiotic crustacean (Zhu *et al.*, 2009). It is also noteworthy that a protein orthologous to LEAM (Tolleter *et al.*, 2007) was found to accumulate in the mitochondria of *Artemia franciscana* encysted embryos (Menze *et al.*, 2009; Hand *et al.*, 2011). It is therefore likely that AMP systematically accumulates in desiccating tissues, thanks to ADK activity, and that maintenance of mitochondrial integrity is a key factor for the achievement of anhydrobiosis in eukaryotes. Finally, while most, if not all, biological processes are expected to be driven by non-equilibrium thermodynamics (Ornes, 2017), the control of energy metabolism during anhydrobiosis offers an original case of a static non-equilibrium. Indeed, the accumulation of AMP in dry tissues guarantees the fast resumption of energy fluxes upon rehydration, just as a pendulum maintained far from equilibrium can restore oscillation upon release.

## Supplementary data

Supplementary data are available at *JXB* online.

Fig. S1. Time course of adenylate species content during imbibition of pea seed fragments at low temperature.

Fig. S2. *In vitro* simulation of the conditions leading to AMP accumulation during seed desiccation and of the adenylate pool equilibration during early rehydration of seed tissues.

Fig. S3. Transcript and protein levels for adenylate kinase genes during Arabidopsis development.

## Supplementary Material

Supplementary Figures S1-S3

## Abbreviations:

ADK: adenylate kinase
AEC: adenylate energy charge.
