# Supplementary material for: Water content, adenylate kinase, and mitochondria drive adenylate balance in dehydrating and imbibing seeds
Source: J Exp Bot. 2017 Jul 20;68(13):3501–12. doi: 10.1093/jxb/erx182 (PMC5853452; doi:10.1093/jxb/erx182)
Supplement: Supplementary Figures S1-S3 [file erx182_suppl_supplementary_figures_s1_s3.pdf]

Raveneau et *al.*

Water content, adenylate kinase and mitochondria govern adenylate balance in dehydrating and imbibing seeds

SUPPLEMENTARY INFORMATION (FIGURE S1, S2, S3)

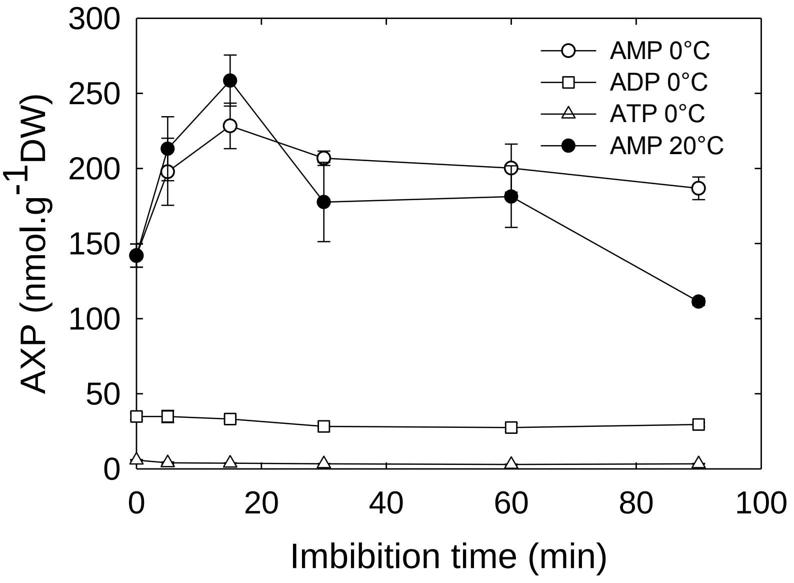

Figure S1. Time course of adenylate species content during imbibition of pea seed fragments at low temperature. Dry pea seeds fragments (approx 500 µm) were allowed to imbibe at 0°C for different times, and adenylates were extracted and quantified by HPLC. The contents of AMP (white circle), ADP (white square) and ATP (white triangle) are indicated, as well as the content of AMP of samples imbibed at 20°C (black circle). Values are indicated with standard deviation (n=3 biological replicates).

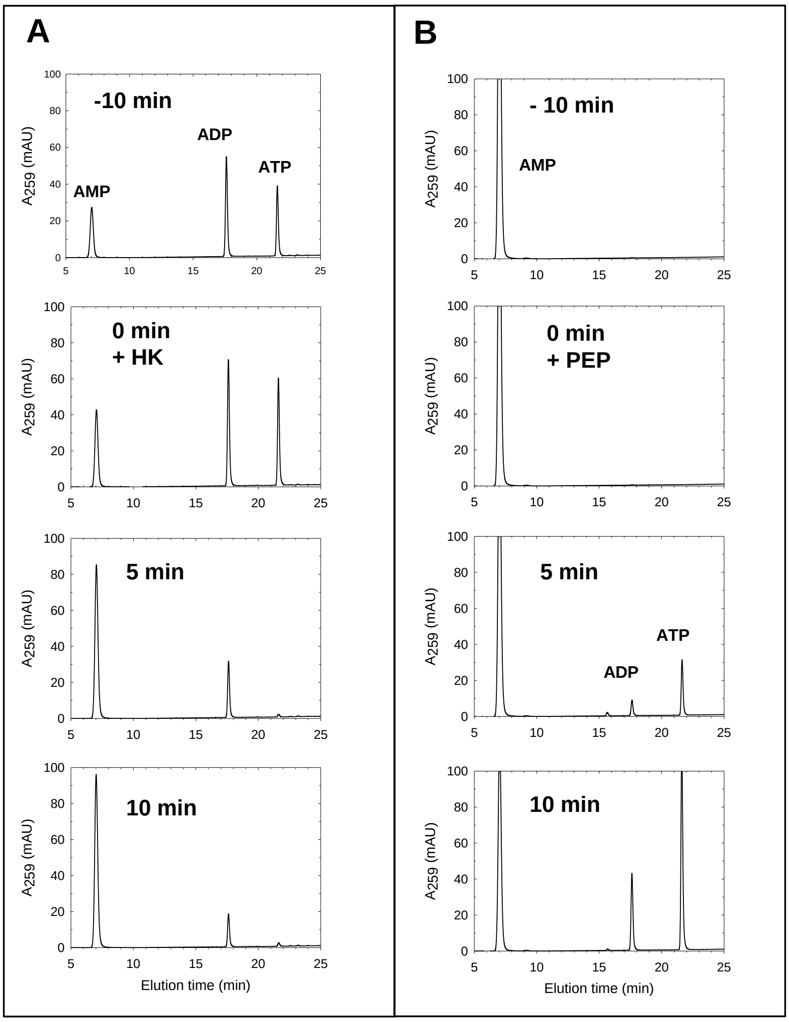

Figure S2. *In vitro* simulation of the conditions leading to AMP accumulation during seed desiccation and of the adenylate pool equilibration during early rehydration of seed tissues. Panel A shows the simulation of AMP accumulation during drying. A mixture of AMP (2.5 mM), ADP (5 mM) and ATP (2.5 mM) was incubated in reaction buffer with 0.5 U of adenylate kinase and 400 mM glucose in a microtube at 30°C. After 10 min, 10 U of hexokinase (HK) were added. Panel B shows the simulation of adenylate pool equilibration during early rehydration. An excess of AMP (20 mM) and a trace of ADP (10 µM) were incubated in a reaction buffer containing 0.5 unit of adenylate kinase and 10 units of pyruvate kinase. After 10 min, 20 mM phosphoenol pyruvate (PEP) was added. Aliquots were directly analyzed by HPLC, and chromatograms are shown for the indicated time of incubation, with the addition of phosphoenolpyruvate as reference.

Transcript levels

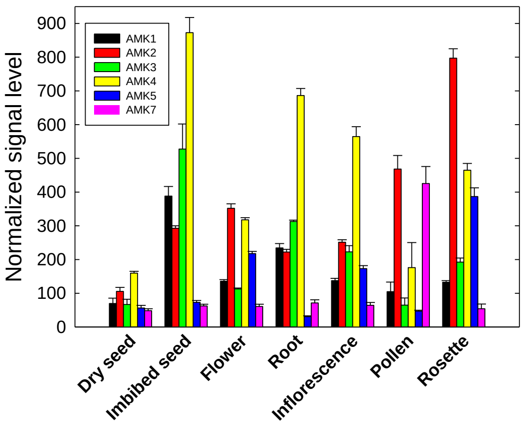

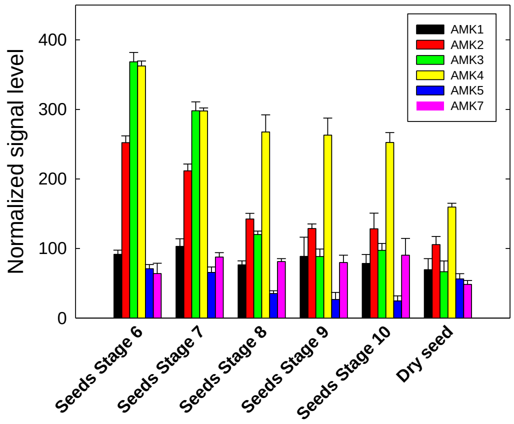

Proteomic quantification

	Flowers	Leaves	Pollen	Roots	Seedling shoots	Seeds	Siliques
AMK1			0.07	0.55			
AMK2	0.48	2.01		0.25	1.79		0.09
AMK3	1.39	0.71	0.94	1.13	0.65	0.38	0.77
AMK4	7.26	4.66	3.10	10.70	2.81	0.08	2.79
AMK5	0.69	1.95			1.33	0.04	0.08
AMK6	0.22	0.03	0.06	0.22			0.21

Fi**gure S3. Transcript and protein levels for adenylate kinase genes during Arabidopsis development.** Absolute values of normalized transcript levels for genes encoding isoforms of adenylate kinase in different tissues were obtained from the Arabidopsis eFP browser (http://bar.utoronto.ca/efp/cgi-bin/efpWeb.cgi). Proteomic data were obtained from the pep2pro database (http://fgcz-pep2pro.uzh.ch/). The values are expressed as normalized spectral counts (single hits were previously removed). Results were color coded as a function of values from yellow to red with Excel. AMK1,At2g37250; AMK2, At5g47840; AMK3, At5g50370; AMK4, At5g63400; AMK5, At5g35170; AMK6, At2g39270; AMK7, At3g01820.
